# Soil carbon and belowground carbon balance of a short‐rotation coppice: assessments from three different approaches

**DOI:** 10.1111/gcbb.12369

**Published:** 2016-06-14

**Authors:** Gonzalo Berhongaray, Melanie S. Verlinden, Laura S. Broeckx, Ivan A. Janssens, Reinhart Ceulemans

**Affiliations:** ^1^ Department of Biology, Research Centre of Excellence on Plant and Vegetation EcologyUniversity of AntwerpUniversiteitsplein 1B‐2610WilrijkBelgium

**Keywords:** bioenergy, carbon fluxes, carbon pools, land‐use change, poplar, *Populus* sp., second‐generation biofuels, soil organic carbon

## Abstract

Uncertainty in soil carbon (C) fluxes across different land‐use transitions is an issue that needs to be addressed for the further deployment of perennial bioenergy crops. A large‐scale short‐rotation coppice (SRC) site with poplar (*Populus*) and willow (*Salix*) was established to examine the land‐use transitions of arable and pasture to bioenergy. Soil C pools, output fluxes of soil CO_2_, CH_4_, dissolved organic carbon (DOC) and volatile organic compounds, as well as input fluxes from litter fall and from roots, were measured over a 4‐year period, along with environmental parameters. Three approaches were used to estimate changes in the soil C. The largest C pool in the soil was the soil organic carbon (SOC) pool and increased after four years of SRC from 10.9 to 13.9 kg C m^−2^. The belowground woody biomass (coarse roots) represented the second largest C pool, followed by the fine roots (Fr). The annual leaf fall represented the largest C input to the soil, followed by weeds and Fr. After the first harvest, we observed a very large C input into the soil from high Fr mortality. The weed inputs decreased as trees grew older and bigger. Soil respiration averaged 568.9 g C m^−2^ yr^−1^. Leaching of DOC increased over the three years from 7.9 to 14.5 g C m^−2^. The pool‐based approach indicated an increase of 3360 g C m^−2^ in the SOC pool over the 4‐year period, which was high when compared with the −27 g C m^−2^ estimated by the flux‐based approach and the −956 g C m^−2^ of the combined eddy‐covariance + biometric approach. High uncertainties were associated to the pool‐based approach. Our results suggest using the C flux approach for the assessment of the short‐/medium‐term SOC balance at our site, while SOC pool changes can only be used for long‐term C balance assessments.

## Introduction

The cultivation of soils with arable crops produces a net carbon (C) flux from the soil to the atmosphere, contributing to the increased greenhouse effect (Le Quéré, *et al*. 2013) and also reducing soil fertility and water quality (Lal, 2004). Afforestation, on the other hand, has been highly recommended to restore C stocks in the soil (Smith *et al*., 1997). Worldwide studies tried to estimate the ability of the soil to sequester C back from the atmosphere (Jones *et al*., 2005; Liang *et al*., 2005; Batjes, 2008; Schulp *et al*., 2008) and to mitigate the (anthropogenic and agricultural) emissions of CO_2_ into the atmosphere.

Short‐rotation coppice (SRC) cultures are defined as high‐density plantations of fast‐growing trees for rotations from 2 to 8 years. At the end of each rotation, the trees are harvested at the base, resulting in the regeneration of new shoots from the remaining stump and the roots. Due to their fast growth and high yield, poplars (*Populus*) and willows (*Salix*) are the most widely used tree species in SRC. Wood chips from SRC biomass can be burned, gasified or co‐fired with coal to produce renewable electricity and/or renewable heat. This type of bioenergy from lignocellulosic feedstock is called second‐generation bioenergy. Therefore, second‐generation bioenergy crops, such as SRC, are considered interesting management options both to sequester C in European croplands, and to partially replace the consumption of fossil fuels (Smith, 2004). However, the cultivation of SRC is more comparable with an arable crop cultivation than with afforestation, despite the woody nature of the planted poplars or willows. Although SRC has been applied since the early 1970s (Hansen *et al*., 1979) as an alternative bioenergy source with a high potential, its capacity to sequester C remains unclear (Walter *et al*., 2015).

Currently, bioenergy is the most significant renewable energy, contributing to almost 80% of the renewable energy supply (IEA, 2014). The European Union is dedicated to increase the amount of renewable energy used to 20% of the total energy consumption by 2020, while simultaneously reducing C emissions by 20% by 2020 (EU 2009). However, there is still a lack of quantitative information on the changes in soil organic C (SOC) for a land‐use change (LUC) to second‐generation bioenergy crops with respect to historical land covers (i.e. arable, grass) and current land management practices (genotypes, planting density, harvest) (Harris *et al*., 2015). Moreover, comprehensive studies on SOC dynamics and greenhouse gas emissions under SRC are limited; subsoil processes and C losses through leaching remain largely unknown (Agosti *et al*., 2015). A large‐scale operational SRC plantation in Belgium, that has been intensively studied within the POPFULL project, provided the opportunity to examine the soil C balance of SRC under the prevailing conditions. The overall aims of this study were to quantify the C balance of the soil of a recently LUC to SRC, and to evaluate the potential of SRC for soil C sequestration. The specific objectives were (i) to close the carbon balance of the belowground compartment of an SRC plantation and (ii) to compare three methodological approaches. As the closure of the carbon balance is a complex and difficult task, we used three different methodologies to reach the primary objective.

## Materials and methods

### Experimental site

The POPFULL project aimed at the full‐system analysis of bioenergy production from an SRC of poplars and involved both an experimental approach at a representative field site and a modelling part (POPFULL project; http://uahost.uantwerpen.be/popfull/). The experimental field site is located in Lochristi, Belgium (51°06′N, 03°51′E), and consists of a high‐density plantation of large monospecific and monogenotypic blocks of both poplar (*Populus* spp.) and willow (*Salix* spp.). Lochristi is located 11 km from Ghent in the province of East‐Flanders at an altitude of 6.25 m above sea level with a flat topography. The long‐term average annual temperature at the site is 9.5 °C, and the average annual precipitation is 726 mm (Royal Meteorological Institute of Belgium). The region is pedologically described as a sandy region and has poor natural drainage; the soil type according the World Reference Base (WRB) is Anthrosol (FAO, 2015). The total area of the site is 18.4 ha. The two former land‐use types of the site were (i) agriculture, consisting of cropland (ryegrass, wheat, potatoes, beets, and most recently monoculture corn with regular nitrogen (N) fertilization at a rate of 200–300 kg ha^−1^ yr^−1^ as liquid animal manure and chemical fertilizers) and (ii) extensively grazed pasture. For more information on the site and the planting scheme, see Broeckx *et al*. (2012) and the Supporting Information (SI).

A detailed soil analysis was carried out in March 2010, prior to planting. The analysis characterized the soil type as a sandy texture. In the upper soil layer, C and N concentrations were significantly lower in cropland as compared with pasture (*P* < 0.05) and decreased exponentially with depth in both former land‐use types. Table 1 presents a detailed analysis of nutrients and soil variables for both land‐use types (see also Broeckx *et al*., 2012).

**Table 1:** Soil bulk density (kg dm^−3^), carbon concentrations (%) and carbon content (kg m^−2^) in the soil organic matter (SOM) at different depths before the planting (2010) and after four years (2014) of short‐rotation coppice culture. As no differences were detected between genotypes (Skado and Koster), data were pooled. The means are presented for both previous land‐use types, and for both narrow and wide rows. Values from narrow and wide rows were averaged taking into account the proportional area they occupied per m^−2^

Depth	2010						2014									
	Cropland			Pasture			Cropland					Pasture				
							Narrow		Wide		Average	Narrow		Wide		Average
	BD	C		BD	C		BD	C	BD	C	C	BD	C	BD	C	C
cm	kg dm^−3^	%	kg m^−2^	kg dm^−3^	%	kg m^−2^	kg dm^−3^	%	kg dm^−3^	%	kg m^−2^	kg dm^−3^	%	kg dm^−3^	%	kg m^−2^
0–15	1.48	1.52	3.37	1.28	1.94	3.73	1.47	1.62	1.49	1.58	3.55	1.47	1.58	1.49	1.65	3.62
15–30	1.44	1.41	3.05	1.42	1.36	2.90	1.12	1.51	1.53	1.49	3.13	1.12	1.90	1.53	1.89	3.96
30–45	1.45	1.06	2.32	1.44	1.18	2.54	1.79	1.43	1.72	1.27	3.45	1.79	1.43	1.72	1.48	3.82
45–60	1.47	0.75	1.66	1.42	1.08	2.30	1.73	1.06	1.78	1.03	2.75	1.73	1.63	1.78	1.22	3.59
60–75	1.57	0.56	1.32	1.46	0.63	1.39										
75–90	1.55	0.34	0.79	1.57	0.36	0.85										

BD, bulk density; C, carbon.

After soil preparation by intensive ploughing (40–70 cm depth), tilling and a pre‐emergent herbicide treatment, a total of 14.5 ha were planted between 7 and 10 April 2010 with 25‐cm‐long dormant and unrooted cuttings from 12 poplar and three willow genotypes in monogenotypic blocks in a double‐row planting scheme with a commercial leek planter (Broeckx *et al*., 2012). The distance between the narrow rows was 75 cm and that between the wide rows was 150 cm. The distance between trees within a row was 110 cm, yielding an overall density of 8000 trees per ha. The total length of individual rows ranged from 45 m up to more than 325 m. Manual and chemical weed control was applied during the first and the second years. Neither fertilization nor irrigation was applied during the entire lifetime of the plantation thus far. Two small portions of the field remained untouched with pasture as unplanted control plots for the determination of soil changes. The plantation was managed in two‐year rotations. After the first rotation (two growing seasons), the plantation was harvested on 2–3 February 2012 using commercially available SRC harvesters (Berhongaray *et al*., 2013b). From there on, trees continued to grow as a coppice culture with multiple shoots for two more years in the second rotation; the second harvest took place on 18–20 February 2014 (Vanbeveren *et al*., 2015). Eddy‐covariance techniques were used to monitor the main greenhouse gases (CO_2_, N_2_O, CH_4_) and recorded continuously from June 2010 till present (Zenone *et al*., 2016). Environmental variables as soil temperature, air temperature, relative humidity, wind speed and precipitations were also monitored. All sensors for these measurements were placed in the immediate proximity of the eddy‐covariance mast (see Data S1).

By the reason of the high labour intensity, and to limit the variability caused by different species and genotypes, only two poplar genotypes were assessed for the soil C and belowground C balance: that is Koster (*Populus deltoides* Marsh* × P. nigra* L.) and Skado (*P. trichocarpa* Hook*. × P. maximowiczii* Henry). Both genotypes were chosen because they are genetically and phenotypically contrasting, and they represented the range of productivity values for the entire plantation (see Broeckx *et al*., 2012 for more details on the productivity of the genotypes).

### Carbon pools

#### Soil organic matter

The C content in the soil organic matter (SOM), known as the soil organic C (SOC), was assessed before plantation establishment (March 2010) and after the second rotation (March 2014). A random sampling was performed at 110 locations in March 2010, of which 60 locations matched with the distribution of the two studied genotypes Skado and Koster, and eight locations with the control pasture. These 68 locations were revisited, and the soil was re‐sampled in March 2014. Half of the 60 locations at the plantation were sampled in each former land‐use type, and within each land‐use type half (i.e. 15) in each of the two row spacings. In March 2010, the soil was sampled up to a depth of 90 cm, while the repeated sampling in March 2014 was only up to 60 cm depth as a previous analysis only showed roots until 60 cm depth (Berhongaray *et al*., 2015). In both campaigns, independent samples were taken every 15 cm on each of the 68 locations using a 2.5 cm diameter and 15 cm length corer (Eijkelkamp Agrisearch equipment, Arnhem, the Netherlands). Bulk density (BD) samples were taken independently using soil BD corer of 5 cm length and with a 5 cm diameter. Carbon mass fractions were determined in three replicates per sample (see below under section *Chemical analysis of soil and biomass samples*). From the C mass fractions and the BD, the C pool per 15 cm depth interval was calculated, and cumulated over 90 cm for the 2010 samples and over 60 cm for the 2014 samples. SOC data were transformed to equivalent soil mass to account for differences in BD between the soil conditions (i.e. previous land‐use type and row spacing). The estimations of SOC at equivalent soil mass were performed for masses of 200, 400, 650 and 900 kg m^−2^ using spline functions as previously described (Berhongaray *et al*., 2013a). For the spline functions, the soil mass was used as the independent variable and SOC as the dependent variable. Interpolations were made by adding or by removing a portion of the soil to reach the desired soil mass assuming that transitions between soil layers were smooth and continuous.

#### Stumps, coarse and medium‐sized roots

Root biomass was determined by excavation of the root system immediately after the two harvests. In February 2012, five trees of different stem diameters (from 20 mm to 60 mm diameter at 22 cm above the soil) were selected within both genotypes (Koster and Skado) for each of both former land‐use types. In February 2014, only four trees per genotype and per land‐use type were excavated. In both excavation campaigns, the remaining stumps and roots were excavated over an area of 1.1 m × 1.125 m (planting distance in the rows x sum of half inter‐row distances). All roots within this area were collected, assuming that roots from adjacent trees compensated for roots of the selected tree growing outside the sampled area (Levillain *et al*., 2011; Razakamanarivo *et al.,* 2012). The excavation depth was limited to 60 cm, as very few roots were observed under 60 cm (Berhongaray *et al*., 2015). Coarse roots (Cr; Ø > 5 mm) and medium‐sized roots (Mr; Ø = 2–5 mm) were sampled; total dry biomass (DM) of these roots (Cr) and of the remaining 15‐cm‐high stump (Stu) was determined after oven drying at 70 °C. As neither a significant effect was found for genotype nor for former land‐use type, all data were pooled. Belowground woody biomass and stump biomass were plotted against basal area, and an allometric regression was fitted. Estimations of the average belowground woody biomass and of the stump biomass pool were made from the diameter inventory of each sampling year, that is from winter 2012 (January 2012) and from winter 2014 (January 2014) as explained in Berhongaray *et al*. (2015). Dried root wood material was grated for C analyses. An average of the C mass fractions was used for calculating the belowground woody C pool.

#### Fine roots

The fine root (Fr, Ø < 2 mm) biomass pool was annually estimated using the soil core methodology. Intact soil samples were taken using an 8 cm diameter × 15 cm deep hand‐driven corer (Eijkelkamp Agrisearch equipment) at the end of each growing season, that is: in winter 2011 (December 2010–February 2011), winter 2012 (December 2011–February 2012), winter 2013 (December 2012), and in winter 2014 (December 2013–January 2014). Winter samples were taken only from the first 15 cm. Samples from different depths were collected during two campaigns in summer, that is: August 2011 and August 2012. In August 2011, sampling was performed in six different soil layers (0–15 cm, 15–30 cm, 30–45 cm, 45–60 cm, 60–75 cm and 75–90 cm, whereas in August 2012 four different soil layers (0–15 cm, 15–30 cm, 30–45 cm, 45–60 cm) were sampled. After each sampling campaign, samples were transported to the laboratory and stored in a freezer until processed. All roots were picked from the sample by hand while (i) separating out weed roots from poplar roots, (ii) sorting poplar roots in dead and living roots, and (iii) sorting poplar roots according to diameter classes (<2 mm and >2 mm). The roots were sorted by visual inspection as previously described (Berhongaray *et al*., 2013c). The sorting of dead (necromass) and living (biomass) roots was based on the darker colour and the poorer cohesion between the cortex and the periderm of the dead roots (Janssens *et al*., 1999). Following washing, fine poplar roots were oven dried at 70 °C for one to four days to determine the standing (fine) root biomass per soil surface area and expressed in g DM m^−2^. More details on root collection and on data processing can be found in Berhongaray *et al*. (2013c,d).

### Carbon fluxes

#### Belowground inputs: fine root productivity and root C input

Sequential soil coring was used to determine Fr mass and Fr production for the second growing season of the first rotation (i.e. 2011) and the first growing season of the second rotation (i.e. 2012). From February 2011 to November 2012, the upper 15 cm of soil layer was sampled every 2–3 weeks (except for the winter when the sampling intensity was decreased) using an 8 cm diameter × 15 cm deep hand‐driven corer (Eijkelkamp Agrisearch equipment). During 2011, 20 samples were collected at every sampling campaign for each genotype. During 2012, the number of samples was different at each sampling date, following the expected intrinsic variability of the Fr biomass based on the experience of the previous year (i.e. 2011). Based on our previously described methodology (Berhongaray *et al*., 2013d), the number of samples in 2012 varied from 12 in winter to 20 in summer. At each sampling campaign in 2011 and in 2012, half of the samples were collected in the narrow and half in the wide rows, randomly distributed over the planted area within the former pasture land‐use type. The samples were transported to the laboratory and stored in a freezer until processed. Once in the laboratory, fine roots were processed as described previously (Berhongaray *et al*., 2013d). Twenty‐one Fr weight of one sample core picked for *x* min (i.e. 5–20 min) was converted into total Fr mass in the sample (i.e. after 60‐min picking duration) using Richard's equation (Berhongaray *et al*., 2013d) and expressed in g DM m^−2^. Subsamples of dried roots were ground for C and N analysis.

For 2011 and 2012 (second growing season of the first rotation and first growing season of the second rotation), root production (*P*) was calculated using the ‘decision matrix’ approach (Fairley & Alexander, 1985). All differences in biomass and necromass were taken into account during the calculation, assuming that the living and dead pools of roots were continuously changing. This approach was better than using the significant differences between root mass of consecutive sampling dates, especially in the case of frequent sampling (Brunner *et al*., 2013), as in our sampling campaign. To calculate annual root production, all productivity values from sampling periods were summed from the beginning until the end of the year. Root productivity calculations and the comparison of different methods were previously described in more detail (Berhongaray *et al*., 2013c).

By the reason of time restrictions, Fr production was estimated with the in‐growth core technique in the second growing season of the second rotation (2013). This method provided reliable estimates for *P* with much less labour time. In December 2012, ten 2.2‐mm mesh bags (10 cm diameter × 0.40 m depth) were installed for each genotype, so 20 in total. Each mesh bag was refilled with root‐free original soil obtained from the root biomass assessment. Root‐free soils were stored in plastic bags, and care was taken to refill the holes with soil with exactly the same stratification. The in‐growth cores were harvested after one year in December 2013. The in‐growth cores were divided into two samples from 0 to 15 cm depth and from 15 to 30 cm depth, and the separated samples were stored in plastic bags until processed. Consequently, only the first 30 cm of the in‐growth cores was used to make it comparable to the 15‐cm increment soil coring approach, and the bottom 10 cm of the in‐growth cores (from 30 to 40 cm) were discarded. The samples were processed in the same way as the samples from the soil coring approach. The *P* was estimated from the quantity of total root mass produced (biomass and necromass) in the considered period of time and expressed in g DM m^−2^ yr^−1^. For periods in which Fr production was not measured, interpolation and extrapolation methods were used. For example, to calculate *P* for 2010, we used the ratio of *P* and Fr biomass from 2011 and the Fr biomass from 2010.

The turnover rate is widely used to estimate root‐derived C inputs to the soil. An assumption of this root turnover approach is that annual Fr production equals fine root mortality on an annual basis. However, the approach of the turnover rate is only valid in steady‐state systems, as, for example mature forests, but not in actively growing systems such as our SRC poplar plantation. In mature forests, the amount of roots produced is the same as those that die at the end of the growing season; they represent the C inputs. In a growing system, part of the productivity is used to form the growing standing biomass. We used the following approach to estimate C inputs from roots (*I*_root_) that consider the increments in root biomass:

(1) $$I_{\mathrm{root}}=\left(P-\Delta Br\right)*C\%$$

where *P* is the root productivity in g DM m^−2^ yr^−1^; Δ*Br* is the difference between root biomass at the end and at the beginning of the growing season in g DM m^−2^ yr^−1^; and C% is the fraction of C (g C g DM^−1^). In our study, this methodology only applied to the fine roots. As a result of the absence of mortality of medium‐sized and coarse roots, productivity of these last mentioned root classes was estimated using Δ*B*, and the C input was equal to zero.

From the in‐growth technique, we obtained evidence for an identical vertical distribution of Fr and root *P*, that is the proportion of *P* at one specific soil depth was similar to the proportion of Fr at the same depth. For years 2011 and 2012, the C inputs from Fr were extrapolated up to 60 cm depth using the measured *P* from the first 15 cm and the vertical distribution of Fr in each year (see above for the fine root depth‐distribution measurements).

### Aboveground inputs

#### Leaf fall

Leaf litter was collected each year (2010, 2011, 2012 and 2013) during the period of leaf fall from early September to December in two plots of 5 × 6 trees for each genotype within each former land‐use type (*n *= 8). In each plot, three perforated litter traps (*i.e*. plastic litter baskets) of 57 cm × 39 cm were placed on the ground along a diagonal transect between the rows covering the wide and the narrow inter‐row spacings. Every two weeks the litter traps of each plot were emptied and leaf dry biomass was determined after oven drying at 70 °C for one week. The collected leaf biomass was cumulated over time to obtain the yearly leaf C input (*I*_leaves_).

#### Weeds and grasses

Before the soil was ploughed in March 2010, the former pasture land was covered by grasses. To account for the C input from these grasses, the aboveground biomass from grasses was harvested in five randomly distributed plots of a contiguous pasture land. Aboveground biomass from weeds was measured after they reached the maximum standing biomass (after flowering) only in two growing seasons, that is: August 2011 and August 2013. In 2011, six randomly distributed plots of 1 m^2^ were harvested under each genotype and from the previous pasture land area. In 2013, four plots of 1 m^2^ were harvested under each genotype and previous land‐use type combination, that is 16 plots in total. In each plot, the weeds were cut at ground level and put in paper bags. The collected weed biomass was oven dried for 10 days at 70 °C and the DM expressed in g DM m^−2^. For years for which we did not measure the weed biomass, we estimated it using the root biomass quantified on these years and the root:shoot ratios from the measured years. The weeds died annually, and the total (weed) biomass was considered as an input to the soil. We estimated the aboveground annual C input from the weeds (*I*_weed_) using the C mass fraction reported in Fortunel *et al*. (2009).

#### Harvest losses

Harvest losses were estimated from samples collected at the field site after both harvests, that is early March 2012 and mid‐March 2014. Two different harvest techniques were used and compared during each harvest, that is two mechanical harvesters in February 2012 (Berhongaray *et al*., 2013b) and a mechanical *vs*. a manual harvesting in February 2014 (Vanbeveren *et al*., 2015). To estimate the harvest losses, harvested woody debris and woody biomass material were collected from the soil surface on four areas of 1 m^2^ within the land area harvested by each harvesting technique for the two genotypes. The collected biomass material and debris were transported to the laboratory and dried in a drying oven at 60–70 °C until constant weight. The harvest losses were expressed in g DM m^−2^, and later expressed as C inputs (*I*_harvest_) using the C mass fraction. More details can be found in Berhongaray *et al*. (2013b) and Vanbeveren *et al*. (2015).

### Carbon outputs

#### Soil CO_2_ efflux

Soil CO_2_ efflux was continuously monitored using an automated soil CO_2_ flux system (LI‐8100; LI‐COR Biosciences, Lincoln, NE, USA) from December 2010 to January 2012 and from May 2012 to January 2014. Sixteen long‐term chambers operating as closed systems were connected to an infrared gas analyser through a multiplexer (LI‐8150; LI‐COR Biosciences). The 16 chambers were spatially distributed over the plantation. Soil CO_2_ efflux was extrapolated for the periods without measurements by a neural network analysis (using MATLAB; 7.12.0, 2011; Mathworks, Natick, MA, USA) based on soil temperature, which was also continuously monitored throughout the year. Values of CO_2_ efflux were integrated over time to obtain the cumulated CO_2_ efflux. More details can be found in Verlinden *et al*. (2013a).

#### Partitioning of soil respiration

To calculate the SOC balance (see below under *Carbon balance*), we quantified the contribution of roots and SOM decomposition to the CO_2_ emission from the soil. The soil CO_2_ efflux (*R*_s_) is the result of CO_2_ release from two main sources: (i) microbial decomposition of SOM (heterotrophic respiration, *R*_h_) and (ii) root‐derived respiration (autotrophic respiration). We partitioned *R*_s_ based on the spatial and the temporal variations in root biomass, in soil temperature, in soil water content and in soil respiration, following the methodology described in Data S3, as follows:

(2) $$R_{s}=R_{h}+R_{m}+R_{\mathrm{gr}}$$

where *R*_m_ is the CO_2_ from the maintenance of root biomass, this rate is assumed to be linearly related to the root biomass to be maintained; *R*_gr_ is the cost of the formation of new root structures and is assumed to be proportional to the growth rate of the roots; *R*_h_ is consequently assumed to be the C output from the SOM pool. The results were annualized and expressed in g C m^−2 ^yr^−1^.

#### Dissolved organic carbon

For the analysis of dissolved organic carbon (DOC) in the soil, 10 groundwater samples were collected monthly from August 2011 until July 2013 from six PVC water tubes (length x diameter: 2 m × 5 cm) distributed randomly under the two genotypes. Water samples were collected using a 2‐m plastic tube connected to a glass bottle by applying a vacuum of 60 kPa. After collection, the samples were stored at 4 °C and sent to an external laboratory (SGS, Antwerp, Belgium) within 24 h. DOC concentrations were determined with a Shimadzu TOC VPH analyser (Shimadzu corp., Japan, 2001) with IR detection after thermal oxidation.

Leaching from the belowground system (see below for a description of the system) was estimated using DOC concentrations and the soil water balance. The soil water balance was calculated as the difference between the monthly cumulative precipitation minus the monthly evapotranspiration, considering positive values as water excess and leaching (Data S4.1). Precipitation was monitored from June 2010 onwards using a tipping‐bucket rain gauge (model 3665R; Spectrum Technologies Inc., Plainfield, IL, USA) installed next to the eddy‐covariance mast (see Data S1). A LI‐7000 fast response gas analyser (LiCor) was used to continuously measure latent heat from air samples at the eddy‐covariance mast from June 2010 onwards. Latent heat flux was converted into evapotranspiration using air temperature and latent heat of vaporization. The annual leaching of DOC was calculated by summing the monthly products of DOC concentrations and water excess. For months without DOC data, the average DOC concentration was used. The annual DOC leaching was also calculated using annual averages of DOC concentration, and annual precipitation and evapotranspiration.

### Chemical analysis of soil and biomass samples

Soil samples as well as dried biomass from wood, leaves and roots were ground and analysed by dry combustion with an NC element analyser (NC‐2100 element analyser; Carlo Erba Instruments, Milano Italy). Soil and plant mass were converted to C mass using the average C mass fraction and expressed in g C m^−2^. The means from different row spacings were calculated separately, and then, the scaled‐up averages were calculated taking into account the proportion of the land area that each row spacing occupied.

### Carbon balance

**Figure 1:**
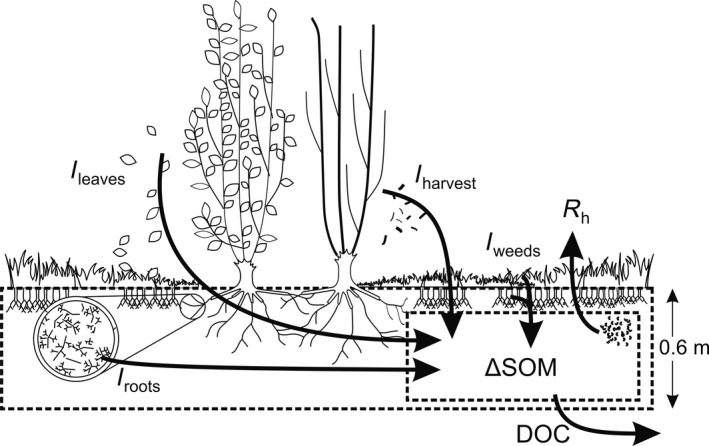
Representation of the soil organic matter (SOM) carbon balance approach. The dashed lines around Δ‐SOM indicate the boundaries that are being considered for the SOM C balance. C, carbon; C fluxes: *I*_leaves_, leaf C input; *I*_roots_, root C input; *I*_harvest_, harvest loses C input; *I*_weeds_, weed C inputs; R_h_, heterotrophic respiration; DOC, dissolved organic C.

The boundaries of the belowground system that we considered for our C balance were the top of the soil surface and a soil depth of 60 cm (Fig. 1). Three different approaches were used to quantify the changes in the SOC, that is (i) the pool change‐based approach, (ii) the component flux‐based approach and (iii) the combined eddy‐covariance + biometric approach. The pool change‐based approach was performed comparing initial and final SOC at equivalent soil mass. The SOC balance was calculated via the component flux‐based approach as:

(3) $$\Delta \text{SOC}=I_{\mathrm{leaves}}+I_{\mathrm{roots}}+I_{\mathrm{weeds}}+I_{\mathrm{harvest}}-R_{h}-\text{DOC}$$

where the C inputs from the different plant components were expressed in g C m^−2^. A few minor components of possible C losses were not measured at the soil level and were thus not taken into account for the SOC balance, that is non‐CO_2_ losses as CO, CH_4_, volatile organic compounds (VOCs) to the atmosphere and herbivory. Moreover, these components only represent a tiny, negligible portion of the soil C emissions (Asensio *et al*., 2007; Görres *et al*., 2015).

Finally, the SOC balance was calculated with the combined eddy‐covariance + biometric approach as:

(4) ΔSOC=NECB−ΔB

where NECB was the net ecosystem C balance representing the overall ecosystem C balance from all sources and sinks – the net ecosystem exchange (NEE, net CO_2_ flux from the ecosystem to the atmosphere); the net CH_4_ efflux; the net efflux; the net DOC leaching loss; and the net lateral transfer of C out of the ecosystem by processes such as anthropogenic transport or harvest; –ΔB is the change in the standing biomass (Stu  +  Cr + Mr + Fr). More details on this last approach were described in detail in Data S5.

### Statistical analyses

Data were analysed with different linear models. A two‐way analysis of variance (anova) was performed using land‐use type and genotype as fixed factors, also including their interactions. More complicated models considered climate, plant and soil variables. These were tested as covariates (*P* ≤ 0.05) and included in the model when significant. In the case of a significant genotype effect, pairwise comparisons were performed using a Tukey's post hoc test (*P* ≤ 0.05). Regression and correlation analyses were performed to search for relationships among variables, the significance of which was tested by an *F* test (*P* ≤ 0.05).

### Uncertainty analysis

The primary obstacles for applying the C balance approach were as follows: (i) the quantification of the annual fluxes of the inputs, the outputs and the changes in the C pools with a reasonable precision, and (ii) the accumulation of errors in the calculation of the C balance as a sum of many components, each with their own error. A combination of error propagation formulas and Bayesian methods as Monte Carlo simulations was used for the uncertainty analysis, following the *IPCC Good Practice Guidance and Uncertainty Management in National Greenhouse Gas Inventories* (IPCC, 2006). The methodology for uncertainty analysis has been explained in detail in Data S6.

## Results

### Carbon pools

As for nearly all terrestrial biomes, the largest C pool in the soil was situated in the SOM. The SOC pool in the first 60 cm of the soil before the planting was on average 10.9 kg C m^−2^ (109 Mg C ha^−1^) vs. 13.9 kg C m^−2^ (139 Mg C ha^−1^) after 4 years of SRC (Table 1). Changes in BD were also observed, especially in the wide rows. Before planting, the vertical distribution of C differed between both land‐use types. In the first layer (0–15 cm), the C% was higher in previous pasture, while in the second layer (15–30 cm), the C% was higher in previous cropland (*P* < 0.05). This vertical distribution was disrupted during the ploughing just before the planting of the SRC. Furthermore, in 2014 the C% was higher in the second layer of the previous pasture as compared to the previous cropland, indicating that the soil was ploughed upside down. The soil layer that was the top layer in 2010 was found in 2014 at a depth of approx. 30 cm. This higher C% is likely a combination of movement of the soil from intensive ploughing and the SRC cultivation for four years. After the conversion to SRC the C% showed a clear spatial distribution, with higher values in the narrow rows than in the wide rows (*P* < 0.05). No differences were found in the control pasture between March 2010 and March 2014 at any depth (see Data S8).

When SOC changes were analysed at the same soil mass (Table 2), we observed a loss of C in the top layer (0–200 kg m^−2^ to 0–15 cm) for the former pasture (*P* = 0.05). In the former cropland, only small, but not significant, C losses were found in the second layer (200–400 kg m^−2^ to 15–30 cm). However, after losses in the first layers, we observed an accumulation of C in the deeper layers for both land‐use types. An overall sequestration of C was found in the entire soil profile (0–900 kg m^−2^ to 0–60 cm) with repeated soil samplings. At equivalent soil masses, the SOC pool in the 0–900 kg m^−2^ (0–60 cm) layer before the planting was on average 11.2 kg C m^−2^ and increased to 14.6 kg C m^−2^ after four years of SRC (Table 2). The higher SOC sequestration was evidenced in the previous pasture land.

**Table 2:** Carbon in SOM at equivalent soil mass before planting (2010) and after four years (2014) of a short‐rotation coppice culture for genotypes Skado and Koster. The difference between 2010 and 2014 (DELTA) is also given. SDs are provided in brackets, and significant differences (anova,* P* < 0.05) between different land uses and years at the same equivalent soil mass are represented with different letters

	Soil mass	2010		2014		Δ	
		Cropland	Pasture	Cropland	Pasture	Cropland	Pasture
	kg m^−2^	kg C m^−2^					
Koster	0–200	3.16 (0.32)a	3.93 (0.29)b	3.30 (0.54)a	3.22 (0.91)a	0.13 (0.62)	−0.71 (0.96)
	200–400	3.10 (1.46)ab	2.05 (0.40)a	3.56 (0.55)bc	4.36 (1.88)c	0.46 (1.63)	2.31 (1.93)
	400–650	3.27 (1.01)a	2.82 (0.90)a	3.87 (1.99)a	3.84 (2.27)a	0.60 (2.25)	1.02 (2.45)
	650–900	2.13 (1.15)ab	1.80 (1.66)a	4.89 (2.24)c	3.93 (5.16)bc	2.76 (2.54)	2.13 (5.43)
	0–900	11.65 (2.23)a	10.61 (2.01)a	15.61 (3.09)b	15.37 (6.01)b	3.95 (3.81)	4.76 (6.33)
Skado	0–200	3.00 (0.59)a	3.79 (0.44)b	3.09 (0.94)a	3.22 (0.72)a	0.09 (1.12)	−0.56 (0.85)
	200–400	3.23 (0.58)a	2.90 (0.33)a	3.19 (1.07)a	4.09 (0.85)b	−0.04 (1.22)	1.18 (0.92)
	400–650	2.74 (1.35)a	3.45 (0.35)a	3.00 (1.38)a	3.79 (1.03)a	0.26 (1.95)	0.34 (1.09)
	650–900	1.15 (1.01)a	2.44 (0.95)b	2.20 (1.72)ab	4.85 (2.73)c	1.06 (2.00)	2.41 (2.89)
	0–900	10.12 (1.91)a	12.58 (1.16)a	11.49 (2.63)a	15.94 (3.13)b	1.37 (3.24)	3.37 (3.34)

SOM, soil organic matter.

The total accumulation of C in Cr, Mr and Stu after four years of SRC was smaller than the changes in SOC (Table 3). The annual change in C stored in the Cr averaged 18.4 g C m^−2^ yr^−1^. This annual change in C was much larger in genotype Skado on the previous cropland, with 22.5 g C m^−2^ yr^−1^, than in the other treatments, which averaged 17.0 g C m^−2^ yr^−1^ (*P* < 0.05). The higher Cr for ‘Skado cropland’ per unit of land area (*i.e*. m^−2^) compared to ‘Skado pasture’ could be explained by the lower tree mortality that resulted in a higher plant density per area (Berhongaray *et al*., 2015). The Mr biomass remained constant between both sampling campaigns, representing about 22% of the total root biomass. No differences were found in Fr between both genotypes (*P* = 0.05). In general, Fr biomass was lower in previous pasture than in previous cropland. Among the plant C pools belowground, the highest amount of C was stored – after four years of SRC – in the woody biomass (Cr and Stu), followed by the Fr and Mr.

**Table 3:** Overview of the belowground carbon pools in the short‐rotation coppice culture: fine roots (Fr), medium‐sized roots (Mr), coarse roots (Cr), stumps (Stu) and soil organic matter (SOM), before planting (winter 2010) and at the end of each growing season (winters 2011, 2012, 2013 and 2014). No differences were detected in Fr for genotypes (Skado and Koster) under both previous land‐use types (cropland and pasture). Fr data were pooled, and the mean and SE (in brackets) are presented. For all other pools, significant differences (anova,* P* < 0.05) were detected; the mean and the range given by the mean values of the combination of genotype*land‐use type are presented. SE, standard error. See Berhongaray *et al*. (2015) for more information on the statistics

	Depth	Fr (0–1 mm)		Fr (1–2 mm)		Mr (2–5 mm)		Cr (>5 mm)		Stu		SOM	
		Mean	SE	Mean	SE	Mean	Range	Mean	Range	Mean	Range	Mean	Range
		g C m^−2^											
Winter 2010	0–15 cm	0.00	–	0.00	–	0.00	–	0.00	–	0.00	–	3473	3260–3700
	0–60 cm	0.00	–	0.00	–	0.00	–	0.00	–	0.00	–	10325	9570–11600
Winter 2011	0–15 cm	4.5	±1.48	1.2	±0.29	–	–	–	–	–		–	–
	0–60 cm	–	–	–	–	–	–	–	–	–	–	–	–
Winter 2012	0–15 cm	14.2	±0.77	7.1	±1.28	18.2	26–65	40.1	27–51	–	–	–	–
	0–60 cm	33.9	–	21.0	–	41.2	74–118	51.9	33–67	129.3	93 –156	–	–
Winter 2013	0–15 cm	10.4	±0.92	6.1	±0.81	–	–	–	–	–	–	–	–
	0–60 cm	24.8	–	12.0	–	–	–	–	–	–	–	–	–
Winter 2014	0–15 cm	22.6	±1.96	13.2	±4.03	19.6	32–68	34.8	32–43	–	–	3242	3170–3330
	0–60 cm	54.1	–	26.0	–	41.4	86–120	73.6	66–90	167.6	152–205	14046	11 000–15 260

### Carbon inputs

The annual leaf fall represented the largest C input to the soil. The total amount of leaf fall increased with the age of the trees, from 2010 to 2013. This C input was exceeded only by the aboveground inputs from weeds in the former pasture land in 2011 and by the Fr in the year 2012, just after the first harvest. After the first harvest, we observed a very high Fr mortality that resulted in a large C input into the soil. During the early stages of land conversion from agriculture to the SRC, annual soil C inputs from weed roots far exceeded those from the poplar trees (Table 4). This was more evident in the former pasture land than in the previous cropland. The contribution of inputs from weed decreased as trees grew older and bigger, while the harvest losses increased. However, the C inputs to the soil after both harvests strongly depended on the operated harvesting machine. The losses during the harvesting reached up to 10.7% of the potential harvestable aboveground biomass (Berhongaray *et al*., 2013b). On average, these C inputs due to the harvest losses were as high as the Fr C inputs.

**Table 4:** Inputs and outputs (release) of carbon (C) from/to the belowground soil system for both previous land‐use types and both genotypes (Koster and Skado). Uncertainties were calculated using Monte Carlo simulations

	Year	Aboveground inputs			SD	Harv	SD	Belowground inputs			SD	Output		DOC	SD	Balance	
		Leaves	SD	Weeds				Weeds	SD	Fr		Rh	SD				SD
Cropland																	
Koster	2010	34	1.43	39	27.8	0	–	32	21.4	58	52.8	312	113.1	7.8	2.5	−156.3	119.8
	2011	72	0.76	54	31.4	45	25.7	175	181.0	73	55.2	314	110.4	9.4	2.0	95.2	164.2
	2012	115	9.74	14	10.1	0	–	85	71.1	125	42.4	285	110.2	12.8	2.8	41.6	137.3
	2013	151	7.82	16	9.7	40	27.8	50	43.2	74	44.0	282	103.6	14.6	3.1	34.5	122.2
Total		372	12.6	124	44.2	85	37.9	341	200.3	331	97.8	1193	218.7	45	5.3	15.0	281.8
Skado	2010	32	11.57	7	4.3	0	–	5	2.8	43	36.3	385	113.6	7.8	2.5	−305.6	118.7
	2011	122	31.05	38	20.4	183	101.6	121	111.3	46	35.9	325	84.1	9.4	2.0	175.4	136.3
	2012	161	46.71	6	4.1	0	–	67	69.7	108	55.3	354	108.3	12.8	2.8	−25.0	134.2
	2013	197	13.96	1	0.7	67	33.3	35	30.3	7	7.1	380	115.9	14.6	3.1	−86.8	123.4
Total		512	58.9	53	21.3	250	106.9	227	134.8	204	75.6	1444	212.4	45	5.3	−242.1	254.9
Pasture																	
Koster	2010	38	4.20	116	63.2	0	–	67	38.6	56	51.5	305	114.2	7.8	2.5	−36.4	144.4
	2011	127	1.93	255	110.4	17	10.6	123	72.2	94	92.0	307	110.5	9.4	2.0	300.2	142.3
	2012	66	32.94	35	20.7	0	–	133	95.0	116	76.5	303	86.1	12.8	2.8	34.0	156.7
	2013	168	20.19	13	9.1	15	9.4	17	17.3	66	50.7	296	99.8	14.6	3.1	−31.8	113.7
Total		399	38.9	418	129.2	32	14.1	340	126.6	332	139.7	1210	206.5	45	5.3	266.1	282.3
Skado	2010	48	15.00	91	52.6	0	–	52	35.9	69	59.4	492	114.4	7.8	2.5	−240.1	171.4
	2011	174	8.99	160	82.4	74	45.7	159	90.2	67	57.8	553	128.3	9.4	2.0	70.2	167.2
	2012	233	40.56	13	8.4	0	–	105	75.2	154	122.2	417	124.9	12.8	2.8	75.7	181.9
	2013	197	36.95	4	2.3	88	50.4	46	41.2	57	51.0	429	131.6	14.6	3.1	−51.8	154.7
Total		652	57.6	268	98.1	162	68.0	362	129.5	346	156.2	1892	249.9	45	5.3	−145.9	337.6

Harv, losses after harvest; Fr, fine roots; Rh, heterotrophic respiration; DOC, dissolved organic C.

All values are in g C m^−2^ yr^−1^.

### Carbon losses

Over the three years of the measurements, *R*_s_ averaged across treatments was 568.9 g C m^−2^ yr^−1^. For all treatments, *R*_s_ was higher in summer than in winter. *R*_s_ continuously increased from 2011 to 2013 in the former cropland, while in the previous pasture, it remained quite stable. Overall *R*_s_ was much higher in the previous pasture and under the genotype Skado. Narrow rows had higher *R*_s_ rates than the wide rows (Data S2). This was related to the higher root biomass in the narrow rows (Berhongaray *et al*., 2013c). The variation in the monthly *R*_s_ was correlated both with soil temperature at 10 cm and with root biomass increment. This allowed to describe the relationship for soil respiration partitioning in root related (autotrophic; *R*_r_) respiration and in *R*_h_. On an annual basis, *R*_h_ accounted from 48 to 79% of the total annual *R*_s_. It ranged from 79% to 95% in winter, and from to 41% to 83% depending on the model used (Fig. S2.4).

We observed a cumulative increase of DOC over the three years of study (2011, 2012 and 2013). The leaching of DOC calculated on a monthly basis increased exponentially from 7.9 (in 2010) to 9.3 (in 2011), 12.8 (in 2012) and 14.5 g C m^−2^ (in 2013). The DOC leaching calculated on an annual basis was a bit lower than on a monthly basis; this was because the calculated water balance on an annual basis was lower than the one calculated on a monthly basis. However, DOC leaching also exponentially increased over the years as presented in Table 4. There was no difference (*P* = 0.05) in DOC concentration between the former land‐use types.

### Carbon balance

**Figure 2:**
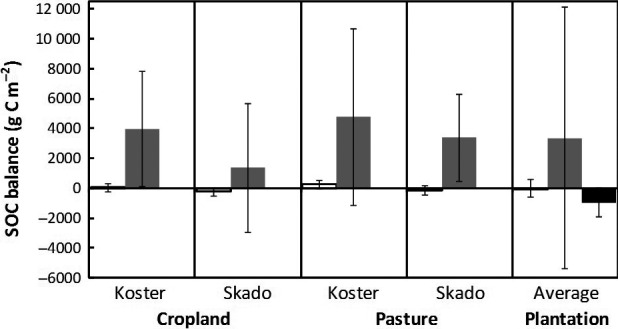
Soil carbon balance using three different approaches where an increased SOC storage is displayed as positive, and a SOC loss is displayed as negative. The left bars represent the component flux‐based approach (non‐filled bars), the central bars represent the pool change‐based approach (bars filled in grey), and the right‐hand bars represent the eddy‐covariance approach. Data from the two contrasting genotypes (i.e. Skado and Koster) and the two land uses (i.e. pasture and cropland) were averaged for the flux‐based and the pool change‐based methods. The combined eddy‐covariance + biometric method represents the SOC change in the plantation, including the two land uses and multiple genotypes. Error bars indicate standard errors of the mean. SOC, soil organic carbon.

Contradicting results were obtained by the pool change‐based approach as compared to the flux‐based and the combined eddy‐covariance + biometric approaches (Fig. 2). The pool change‐based approach resulted in an average SOC increase of 4360 g C m^−2^ for genotype Koster and 2370 g C m^−2^ for Skado. However, the flux‐based approach resulted in a small increase of 140 g C m^−2^ for genotype Koster and a small decrease of −194 g C m^−2^ for Skado. The main C inputs to the soil resulted from the leaf litter fall, from annual weeds and fine roots (Table 4). The total C inputs over the four years ranged from a potential minimum of 730 g C m^−2^ to a potential maximum of 1530 g C m^−2^ depending on the genotype, on the previous land‐use type and on the used harvesting machine. The main C flux released from the soil came from soil respiration; the leaching of DOC represented only a very minor proportion (<3%). The total C released from the soil ranged from 1193 g C m^−2^ to 1892 g C m^−2^ for the four years. If we added the C stored in the woody biomass pools, the belowground system resulted in a net gain of C after four years of SRC in both genotypes and both former land‐use types. However, with the combined eddy‐covariance + biometric approach – which integrated over different genotypes and land uses – net C losses of −956 g C m^−2^ were estimated over the four‐year period.

### Uncertainties

Uncertainties in the SOC balance were highest in the pool change‐based approach, followed by the eddy‐covariance + biometric and the flux‐based approaches (Fig. 2). Although the estimations with the pool change‐based approach were highly sensitive to the BD data, most of the uncertainty was on the C% data, contributing to 82% of the uncertainty. Among the seven variables included in the eddy‐covariance + biometric approach, the harvested biomass (see Fpc in Data S5) was the most sensitive and contributed to 84% of the uncertainty. In the flux‐based approach, the degree of uncertainty was not related to the size of the flux. In this last mentioned approach, most of the uncertainty was on the belowground fluxes (Table 4). Rh estimations contributed with 32% to the uncertainty, followed by the weeds and fine root inputs with 38% combined. While uncertainties from the aboveground inputs were relatively low, the uncertainty was reduced by on average 18% with the use of Monte Carlo simulations as compared to the simple error propagation formulas (data not shown).

## Discussion

### Belowground pool and fluxes, and SOM C balance

Our pool‐based approach indicated an average increase of 3360 g C m^−2^ (or 33.6 Mg ha^−1^) in the SOM pool, which is a large value when compared with the flux‐based and the combined eddy‐covariance + biometric approaches (Fig. 2). High accumulations of SOC were found deep in the soil (below 30 cm depth), but small gains of C were also measured in the top layer. The soil was ploughed upside down before planting, putting C‐rich soil soil deep in the soil where SOC decomposition processes were reduced due to low temperature, low priming effects (low root exudates) and frequent soil saturation by the water table. On the other hand, initially deep low‐C soil was placed in the top layer, where most C inputs occurred. This initial low‐C soil had a high potential to form soil structures and physically protect new SOC from decomposition. These two mechanisms for the top and the deep SOC might explain the C increases in the soil. Other studies also showed that soil depth significantly influenced SOC change rates and should thus be considered in C emission accounting in SRC cultures (Qin *et al*., 2016). The belowground woody biomass (Stu, Cr, Mr) represented the second largest C pool of the SRC. This long‐term belowground biomass also contributed to enhance the C sequestration along the four‐year sequence (Pacaldo *et al*., 2014). The value observed for the belowground biomass C sequestration (240 g C m^−2^) was much higher than the 90 g C m^−2^ reported for an SRC plantation in Canada (Arevalo *et al*., 2011). This might be explained by the higher planting density at our site.

Although not all fluxes were continuously measured, especially in the former cropland, we were able to identify and quantify the main fluxes. Our estimates of the SOM C balance depended on the genotypes, on the weed control, and on the harvesting machines (Table 4). In the future selection of the appropriate management, the choice of the suitable genotype, the process of weeding and the efficiency of the harvesting process are all important for the SOC sequestration.

### Effect of the previous land‐use type

The flux‐based C balance was lower in the previous cropland than in the former pasture land. This was explained by the higher C inputs in the former pasture. These higher inputs in the former pasture came in particular from leaf fall and from weeds. However, the C inputs were measured with less intensity (fewer locations and occasions) in cropland. This might have slightly altered the C balance in favour of the previous cropland. Nevertheless, the pool‐based approach also showed higher accumulations of SOC in the former pasture land.

Changes of the total SOC pool as a result of land‐use change from cropland and pasture to SRC in Central Europe were recently reported (Walter *et al*., 2015) and ranged from −1.3 to 1.4 Mg C ha^***−***1^ yr^***−***1^ (converted from cropland) and from −0.6 Mg C ha^***−***1^ yr^***−***1^ to *+*0.1 Mg C ha^***−***1^ yr^***−***1^ (converted from pasture). Overall, there was no SOC change in the study of Walter *et al*. (2014) which is in line with results of a 20‐year chronosequence for SRC plantations in the USA (Pacaldo *et al*., 2013). These findings suggest that the C inputs from short‐term components (as Fr, leaves, weeds) did not result in a SOC accumulation over time. In contrast, a chronosequence of SRC cultures in Canada showed that soils initially lost C while after two years soil C levels increased and reached the initial values in the seventh year (Arevalo *et al*., 2011).

#### Effects of harvesting and of the presence of weeds

Our study showed that harvesting played an important role in the soil C balance. Overall, the inputs from harvest losses were as high as the Fr inputs. The C inputs from the harvest losses were higher in the former cropland, which can possibly be explained by the higher aboveground biomass productions (and yields) in the cropland. This demonstrated that the harvesting operation had an effect on the C balance of the system. However, the harvest losses have negative implications on the energy production; the more biomass that is left in the field, the less there is for energy generation. Litter fall is temporarily reduced in frequently harvested tree plantations (Jandl *et al*., 2007); this reduces the C inputs and contributes to lower soil C stocks. The input of harvest losses into the soil may compensate for the smaller litter fall inputs. Additionally, we found an increased belowground input from Fr mortality after harvest. Apart from the changed C inputs, the harvest might have secondary effects. For example, harvesting changes the microclimate. Decomposition of forest floor C is temporarily stimulated after harvest, because the soil becomes warmer and possibly wetter due to the reduced evapotranspiration (Piene & Vancleve, 1978). Moreover, a harvested field is more exposed to wind and to erosion. Field studies in timber plantations showed that SOC decreased with increasing harvest intensity (Nave *et al*., 2010). We found very high annual C inputs from weeds, especially in the first rotation. Annual grasses can offset the removal of C inputs in bioenergy crops by providing additional biomass and thus C input (Blanco‐Canqui, 2013).

#### Soil CO_2_ efflux

*R*_s_ constituted the largest flux to return belowground C to the atmosphere, and it represented the combined *R*_r_ and *R*_h_. The *R*_s_ represented 55% of the total ecosystem respiration in our SRC (Verlinden *et al*., 2013b), with roots representing about 47–79% of the total *R*_s_ (Data S2). The current study revealed a large *R*_s_ during the four years of SRC, ranging from 470 to 785 g C m^−2^ yr^−1^. These values are within the range of *R*_s_ values of 740–970 g C m^−2^ yr^−1^ obtained in different willow SRC plantations in the USA under a similar planting scheme and comparable climatic conditions as our plantation. Other measurements of *R*_s_ on poplar SRC plantations were recorded over shorter time periods and are not comparable (Arevalo *et al*., 2011).

In the former cropland, there was an increasing *R*_s_ throughout the years. This might contradict results from other SRCs where *R*_s_ remained rather constant over the years after agricultural lands were converted to SRC (Arevalo *et al*., 2011). However, this increase was not observed in the previous pasture land. The higher *R*_s_ in the pasture compared with the cropland might be attributed to the higher initial SOC in previous pasture and to the higher root biomass and growth of genotype Skado (Verlinden *et al*., 2015).

#### Doc

The annual DOC leaching increased exponentially throughout the years, and this was driven by the water balance. With regard to our DOC measurements, very similar annual estimates (7–13 g C m^−2^ yr^−1^) were reported for forests in Belgium (Gielen *et al*., 2011) and in Germany (Borken *et al*., 2011). Moreover, for forests (Gielen *et al*., 2011) as well as for agroecosystems (Brye *et al*., 2001) the interannual variability of DOC fluxes is primarily driven by the water balance, in line with our observations.

### Uncertainties

The uncertainties in the quantification of the C pools, of the C inputs and of the C outputs have different sources: (i) the measurement error involved in the data collection, and (ii) the prediction error, when a model was used for predictions. The measurement errors were due to the intrinsic variability of the measured variable and to errors in the measurements. Furthermore, in our SRC plantation spatial variability was generated by the double‐row planting system, by the different genotypes and by the previous land‐use types. The prediction errors resulted from (i) the model itself, through its error term and the uncertainty of its parameters and (ii) the uncertainty of the other variables used in the model (Molto *et al*., 2013). The use of combined error propagation formulas and Monte Carlo simulations allowed a proper treatment of the uncertainties.

We were able to quantify the uncertainties in the estimation of the soil C balance, as well as the contribution by the various input variables to these uncertainties. Some variables showed a very low uncertainty (including a low standard deviation), as they were measured in a small area; the representativeness of these values was difficult to quantify. On the other hand, some input variables contributed only very little to the overall uncertainty; they represented a very low input value, but the uncertainty on these variables might be very large. Below, we review and discuss the uncertainties on the estimation of each input variable itself. This is relevant for future research to improve the estimates of key input variables.

### Uncertainties in the flux‐based approach

In general, soil characteristics are highly spatially variable over short distances. A high degree of uncertainty is created by the low capture of the spatial heterogeneity in the *R*_s_ estimations. The measurements of *R*_s_ were concentrated on a rather small area of the plantation because of various logistic reasons, as the restricted length of the instrument cables and the necessity of mains power supply (Verlinden *et al*., 2013). Moreover, the proportion of *R*_h_ to *R*_s_ was high. The contribution of *R*_h_ has been estimated to be between 10 and 90% of *R*_s_ (Hanson *et al*., 2000), with an average of 60%. Our models predicted the proportion of *R*_h_ within the range of previous studies, but close to the higher values. The other variables were measured over a larger area of the plantation and might have a lower spatial uncertainty. Uncertainties were also created by the upscaling models, by the calculation methods, etc. For example, the uncertainties associated with our estimations of the DOC leaching highly depended on the water balance estimation. Uncertainties in the estimations of Fr productivity were associated with the method used (Berhongaray *et al*., 2013c), as well as with the *R*_s_ partitioning (Data S2). On the other hand, mycorrhizal inputs were not quantified at our plantation. Mycorrhizal inputs are a dominant process for C input in poplar plantations (up to 68% of the total inputs), exceeding the input via leaf litter and fine root turnover (Godbold *et al*., 2006). Aboveground inputs from weeds were also subject to a high uncertainty. This high uncertainty was created by the high spatial heterogeneity and the rather low sampling intensity and frequency. Due to time constraints and logistic management issues, aboveground weed biomass was measured with few replicates, that is only in two of the four years of the study, and only once in the previous pasture land area. The proper assessment of the uncertainties with the Monte Carlo simulation allowed the reduction of the uncertainty in the multivariable flux‐based SOC balance.

### Uncertainties in the pool‐based approach

For the SOC determination, we captured the spatial heterogeneity. A strong determinant in the change of the SOC stock was the change in BD. The values of BD for March 2014 were too high for the soil below the 30 cm depth. Large uncertainties were associated to the BD estimations at these depths. However, the high BD values below the 30 cm depth were related to the high soil compaction measurements at the same depth (see Data S2), concluding that even with large uncertainties, the mean values were reasonable. If an average BD of 1.5 was used instead of 1.76, the SOC change would be half, which still represents a large positive SOC change. Moreover, the soil was a deep ploughed Anthrosol soil. Due to the deep ploughing, a high spatial heterogeneity of SOC has been induced. This is visible in Table 2 in the increasing standard deviation from 2010 to 2014. Also the more increased SOC pools in the subsoil layer as compared to the topsoil indicated a strong deep ploughing soil inversion effect. To reduce the uncertainty in the estimation of the pool change‐based approach, we used a control pasture. No changes in SOC were found in the control grassland. This allowed to attribute all the SOC changes to the changes in the land use, and not to methodological or climatic reasons. Taken all considerations into account, the pool‐based approach reflected an unrealistically high SOC change, limiting its use in the evaluation of short‐term SOC changes.

### Uncertainties in the combined eddy‐covariance + biometric approach

The eddy‐covariance measurements, including CO_2_ and CH_4_ fluxes, probably had a high degree of uncertainty due to the size of the footprint. Usually, the size of the footprint increases with changes in atmospheric stability from unstable (day) to stable (night) conditions, directly affecting the NEE estimations (Leclerc & Thurtell, 1990). DOCs and VOCs were measured with a smaller frequency or during shorter periods, but they only represented a tiny portion of the C balance and their impact on the uncertainty was small. The uncertainties on the harvested biomass were rather low. The total biomass yield of the site was recently quantified using three different methodologies with a very good agreement among them (Verlinden *et al.,* 2015).

The highly spatial variability together with the high resilience dynamics of the SOC stocks require long (>20 years) periods to quantify SOC changes using the pool‐change approach (Guo & Gifford, 2002). On the other hand, medium‐term (2–5 years) flux measurements can account for SOC changes, and provide better estimates about whether the soil pool or reservoir is functioning as a source or as a sink for C. Our results showed a small C increase in the belowground compartment of the SRC plantation. However, results from the entire life of an SRC (around 20 years) should be considered to substantiate the C storage potential of this type of bioenergy crop. Unfortunately, C sequestration in the soil is not permanent and it seems that forest soils are reaching an equilibrium (Janssens *et al*., 2005). Compared to the reduced emissions of other GHG sources, which can continue indefinitely, C sequestration in the soil is therefore time‐limited and finite. This limitation is explained by the sink saturation (Stewart *et al*., 2007) and because further increases in forest areas are unlikely (Jandl *et al*., 2007).

The additional heterogeneity made SOC pool assessments much more difficult than in common soils. So, the conclusions on the best choice of methods are limited to our site. Regardless of the soil C sequestration, the C fixation in bioenergy crops provides large benefits reducing CO_2_ emissions which make bioenergy crops useful and beneficial. The results presented in this study are of high relevance for bioenergy crop models and for C stock estimations. Life cycle analysis studies of SRC for bioenergy will also benefit from this and similar soil C balance assessments. This information is also crucial for policymakers for the proper evaluation and further improvement of this renewable source of energy.

## Supporting information


**Data S1.** Geospatial distribution of the field site.
**Data S2.** Bulk density.
**Data S3.** Partitioning soil respiration.
**Data S4.** DOC Leaching.
**Data S5.** SOC balance using the eddy‐covariance+biometric approach.
**Data S6.** Error estimation and Uncertainty analysis.
**Data S7.** Models used for statistics.
**Data S8.** Soil C in control plots.
