# Supplementary material for: Soil carbon and belowground carbon balance of a short‐rotation coppice: assessments from three different approaches
Source: Glob Change Biol Bioenergy. 2016 Jun 14;9(2):299–313. doi: 10.1111/gcbb.12369 (PMC5310368; doi:10.1111/gcbb.12369)
Supplement: Supplementary file 1 — Data S1. Geospatial distribution of the field site. Data S2. Bulk density. Data S3. Partitioning soil respiration. Data S4. DOC Leaching. Data S5. SOC balance using the eddy‐covariance+biometric approach. Data S6. Error estimation and Uncertainty analysis. Data S7. Models used for statistics. Data S8. Soil C in control plots. [file GCBB-9-299-s001.docx]

**Supporting Information**

# GCB-B-OR-15-178

***S1: Geospatial distribution of the field site***


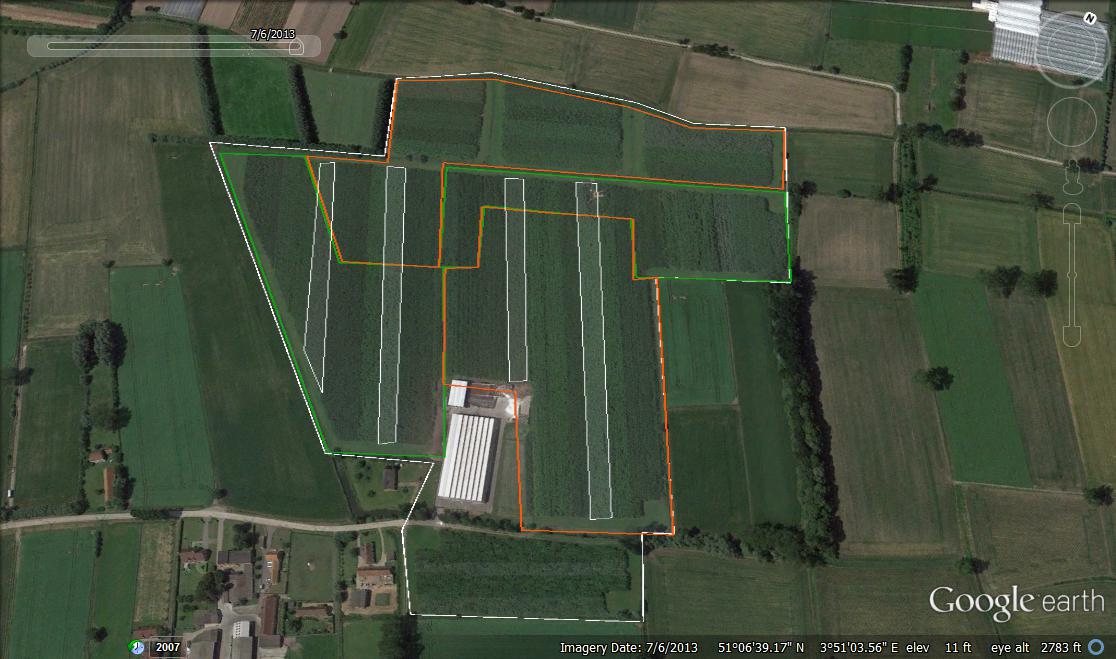


Figure S1: Aerial image of the field site before and after the establishment of the SRC. The map shows the distribution of the previous land-use types, i.e. pasture land (green lines) and cropland (orange lines), and the monoclonal blocks (white lines) indicating the location of genotypes Skado and Koster. Use the application to move through the dates and see the changes in land use. (Source: Google Earth)

***S2: Bulk density***

For purposes of verification with independent data, we compared the bulk density (BD) data with soil compaction measurements (Vanbeveren et al., 2015). We assessed the soil compaction through measurements of the pressure needed to penetrate the soil with a penetrologger (Eijkelkamp type 06.15.SA, The Netherlands). The procedure as described in the instrument manual was followed with a 1 cm^2^ cone surface area. As an output, a graph was generated, showing a pressure profile with depth. We randomly measured 36 transects with eight sampling points in each transect, equally spread over monoclonal blocks of two genotypes, i.e. Skado and Koster. From the eight sampling points, points 1-3 were located in and averaged as a measure for the narrow row, as was done for points 4-8 for the wide row. The wide rows are used for transit of agricultural vehicles and the narrow rows can be seen as control rows. Measurements narrow vs. wide rows were averaged, resulting in two curves (Fig. S2.1).


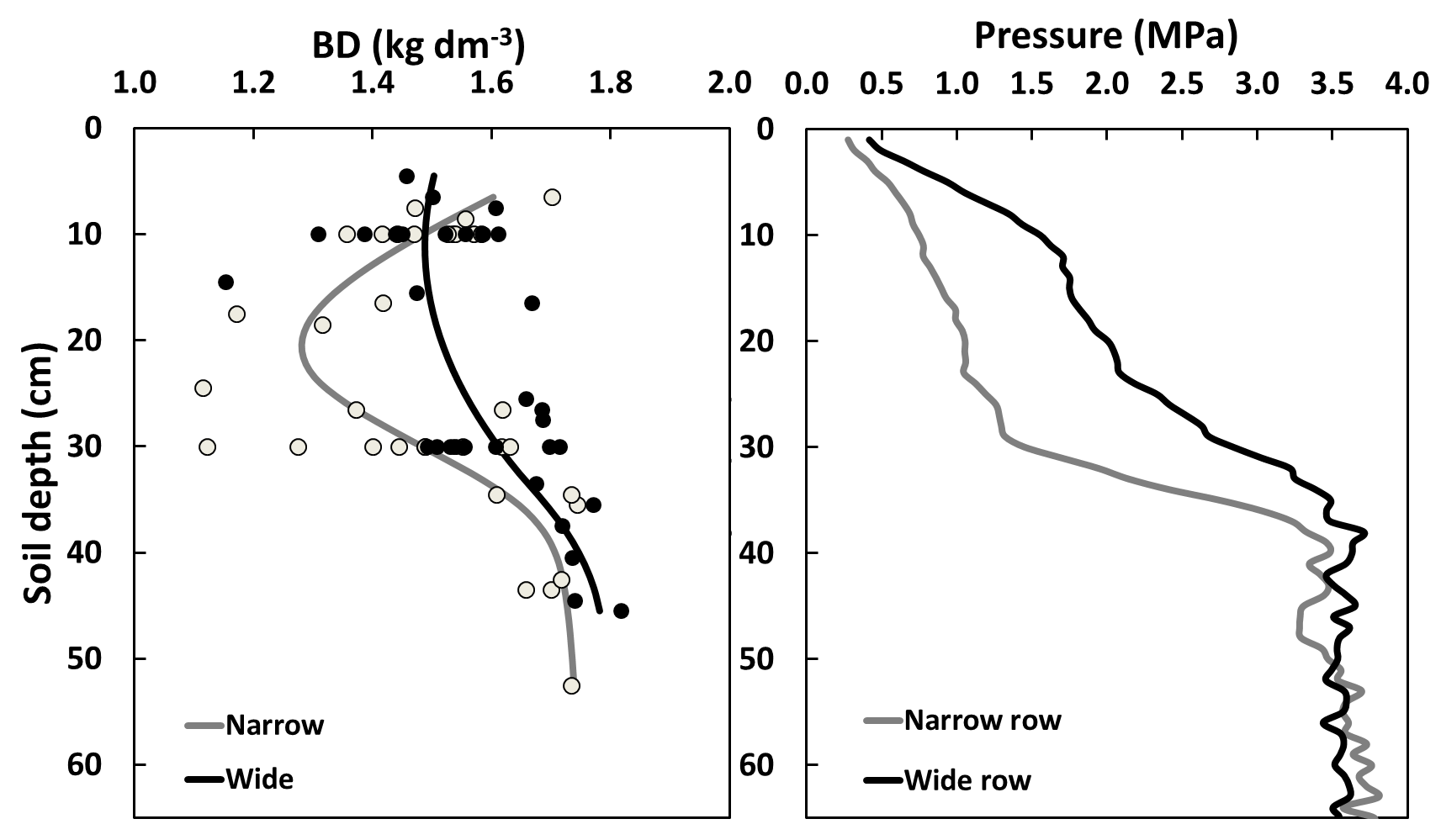


S2.1: Depth distribution of bulk density (BD) and soil compaction (Pressure) in the narrow and wide rows.

We observed a rapid increase in soil compaction in the 0-10 cm layer (Fig. S2.1), representing the presence of a hard soil in this top layer. These measurements coincided with high BD values in the same layer. From 10 to 30 cm depth, the penetrometer needed less pressure to penetrate the soil, meaning the presence of a “soft” soil layer, coincident with low BD values. Below 30 cm there is a very hard-to-penetrate soil layer, equivalent to the very high BD values in the deep soil. These two independent measurements were confirmed the highly variable BD measurements. Soil compaction measurements were done in more than 280 profiles, with a good spatial distribution, while BD was measured at 24 profiles only.

***S3: Partitioning soil respiration***

We used a non-invasive method for R_s_ partitioning, in which the relationship between R_s_ and root biomass was extrapolated to the intercept, yielding R_h_ (Rodeghiero and Cescatti 2006; Xu et al. 2001). This is an indirect way for partitioning R_s_ that provides an approximate quantification of the contribution of roots to total soil respiration (Baggs 2006). However, this method used the regression technique which did not take the seasonal evolution of belowground processes into account, as root growth and seasonal changes in root biomass. We used chamber techniques to measure R_s_, and we took advantage of *in situ* spatial differences in fine root biomass and production, and in R_s_ (Berhongaray et al., 2013c; Verlinden et al., 2013b) to fit non-linear models and capture seasonal changes in R_h_.

Half of the 16 chambers for soil respiration (R_s_) measuremets were installed in the narrow inter-rows, the other half in the wide inter-rows (Verlinden et al., 2013b). Meteorological and soil variables were recorded half hourly at the site in one location *ca.* 30 m away from the location of the soil CO_2_ measurements. Soil water content (W_s_, m^3^ m^-3^) was measured using a probe (TDR model CS616; Campbell Scientific) placed at 20 cm depth. Soil temperature (T_s_) at 0-10 cm depth was measured using a thermocouple (model TCAV-L, Campbell Scientific, Logan, UT, USA) every 10 s and the 30 min averages were stored on a data logger. Mean W_s_ and T_s_ were calculated for the period between sampling dates. From 22 February 2011 to 6 November 2012 the upper 15 cm soil layer was sampled approximately every two weeks - a total of 33 sampling campaigns (Berhongaray et al., 2012).

The growth pattern of coarse roots (Cr) during the growing seasons was simulated using a sigmoid function. Since it was not possible to monitor the evolution of Cr biomass through the growing season and since most growth processes follow a sigmoid curve, we used the evolution of leaf area index (LAI) to estimate the Cr growth (Fig. S2.1). The LAI was a reliable descriptor of aboveground growth at the site (Broeckx et al., 2013), and we assumed that the above- and the belowground growth occurred at the same time and showed the same pattern. The LAI data were transformed to cumulative LAI values and then different sigmoid functions were fitted to the increment of cumulative LAI with time (Fig. S2.1). The best fit was obtained with the cumulative symmetric double sigmoid function:

[Eq. S3.1]


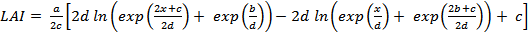

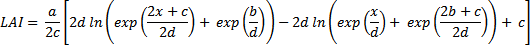


where *x* is the day of the year, *a* represents the transition magnitude or height, *b* represents the midpoint of the transition *c* controls the width of the transition, and *d* controls the shape of the transition.

Figure S3.1: Seasonal evolution of leaf area index (LAI, left panel) and the cumulated values of LAI (right panel) of the poplar plantation during the second growing season (of the first rotation) i.e. 2011. The line in the right panel represents the sigmoid fit to the data: Eq. 1, a= 22.95, b=154.1, c= 231.4, d= 5.053 (after Broeckx et al. 20112b).

Cr growth was then estimated as a function of cumulative LAI, using Eq. S3.1. For the Cr growth over time, parameter *a* was replaced by the Cr biomass at the end of the growing season, and the initial Cr was added to the estimations.

The study period included 33 sampling campaigns from 1 January 2011 until 6 November 2012, that corresponded with the 33 sampling campaigns of Fr. For the periods between the 33 root sampling campaigns we calculated the mean F_r_ biomass and productivity, as well the mean C_r_ biomass; we cumulated R_s_ and we averaged the hourly data T_s_ and W_s_, in order to obtain one value per variable per period. Based on the spatial and the temporal variation in root biomass and soil respiration we partitioned R_s_ into heterotrophic (R_h_) and root derived respiration (R_r_):

*R_s_ = R_h_ + R_r_* [Eq. S3.2]


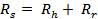


The rate of CO_2_ produced by the roots and by the rhizosphere (R_r_) was further separated into two major components of respiration: the maintenance (R_m_) and the growth component (R_gr_):

*R_r_ = R_m_ + R_gr_* [Eq. S3.3]


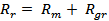


R_m_ is the CO_2_ production for the maintenance of root biomass; this rate is assumed to be linearly related to the root biomass to be maintained. R_gr_ is the cost associated to form new root structures, and is assumed to be proportional to the growth rate of the roots. Only poplar fine root biomass was used for the partitioning, since weeds were manually removed from the soil chambers (see in the paper the S*oil CO_2_ efflux* section). The root growth rate (Gr) was determined as the slope of the total root biomass increment (*Br*) between two sampling dates of the 33 Fr sampling campaigns versus the time (*t*) between those sampling dates:

| *Gr =* | *ΔBr* |
| --- | --- |
|  | *Δt* |


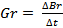


[Eq. S3.4]

R_s_ was then calculated for each sampling period by cumulating the values over time since the previous sampling date and decomposed in three major components:

*R_s_ = R_h_ + R_m_ + R_gr_* [Eq. S3.5]

We used non-linear models to estimate R_s_ based on the spatio-temporal variation in root biomass and root growth, and the temporal variation in soil temperature (T_s_) and in soil water content (W_s_). To build the non-linear models we used information and relationships previously published in the literature. We therefore used the same parameter (β_2_) to describe the sensitivity of R_h_ and R_m_ to T_s_. As a result of the relationships described above in the introduction, we used the following two equations:

*R_h_ = β_1_ * e^Ts * β2^* [Eq. S3.6]


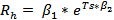


R_m_ = *β_3_ * B_r_ * e^Ts * β2^* [Eq. S3.7]


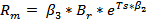


The respiration derived from growth is linearly related to the growth rate (Scheurwater et al., 1998) as follows:

R_gr_ = *β_4_* * G_r_ [Eq. S3.8]


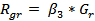


We designed non-linear models that either considered or not the W_s_ effect, and both the multiplicative as well as the additive effect of W_s_ to the exponential response of T_s_ (Curiel-Yuste et al., 2003). In line with the literature, and considering equations [A1.6], [A1.7] and [A1.8] above, we designed the following three non-linear models for partitioning of R_s_:

*R_s_ = β_1_ * e^Ts * β2^* + *β_3_ * B_r_ * e^Ts * β2^* + *β_4_* * G_r_ [Eq. S3.9]


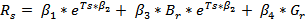


*R_s_ = β_1_ * W_s_ * e^Ts * β2^* + *β_3_ * B_r_ * e^Ts * β2^* + *β_4_* * G_r_ [Eq. S3.10]


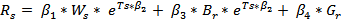


*R_s_ = β_1_ * e^Ts * β2^* + *β_3_ * B_r_ * e^Ts * β2^* + *β_4_* * G_r_ + *β_5_* * W_s_ [Eq. S3.11]


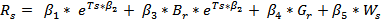


The R_h_ component was calculated by setting B_r_ and G_r_ to zero; the R_r_ component was determined as the difference between R_s_ and R_h_.

The optimization of the non-linear models was done following the downhill simplex method of Nelder and Mead (1965). The significance of the variables and of the parameters was determined. Slopes and intercepts of predicted vs. observed data were compared by the t-test (Fila et al., 2003). Root mean square error (RMSE) (Kobayashi and Salam, 2000) was calculated for each estimation methodology and we evaluated the trade-off between the goodness of fit of the model and the complexity of the model using the corrected Akaike information criterion (AICc) (Burnham and Anderson 2002):

| *AICc = – 2 ln(L) +* | *2k (k + 1)* |
| --- | --- |
|  | *n – k – 1* |

[Eq. S3.12]

where *k* is the number of parameters in the model, *L* is the maximized value of the likelihood function for the estimated model, and *n* is the number of samples.

We observed that throughout the growing season, B_r_ was higher in the narrow rows than in the wide inter-rows. In general, the pulses of G_r_ were also higher in the narrow rows. Together with the temporal differences in T_s_ and W_s_ these spatial differences in B_r_ and G_r_ allowed us to fit the non-linear models.

Figure S3.2: Seasonal evolution of soil temperature, soil water content, root biomass and root growth during 2011 and 2012 for Skado in pasture land-use. White squares represent the root biomass and root growth in the narrow inter-rows, and the black squares are those in the wide inter-rows.

Table S3.1: Simplified root respiration model of short-rotation coppice and the values of the estimated parameters. Adjusted parameters for heterotrophic, root maintenance and root growth respiration estimations, and p-values are shown. The root mean square error (RMSE) and the corrected AIC (AICc) of each model are also provided.

| Model | Syntax | Parameters | | | | | RMSE | AICc |
| --- | --- | --- | --- | --- | --- | --- | --- | --- |
|  |  | 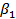 (p-value) | 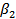 (p-value) | 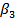 (p-value) | 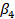 (p-value) | 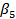 (p-value) |  |  |
| Model 1 | [Eq. 9] | 0.181 (<0.001) | 0.162  (<0.001) | 0.00132 (0.003) | 0.0312 (0.318) |  | 0.846 | 244.63 |
| Model 2 | [Eq. 10] | 0.284 (0.011) | 0.192 (<0.001) | 0.000202 (0.013) | 0.0577 (<0.217) |  |  | 337.65 |
| Model 3 | [Eq. 11] | 0.331 (0.005) | 0.131  (<0.001) | 0.000231 (0.018) | 0.0246 (0.433) | -1.36 (0.101) |  | 244.03 |

On an annual basis, R_h_ accounted from 48 to 79% of the total annual R_s_. It varied from 95%, 84% and 79% in the first winter, and to 83%, 41% and 83% in summer for models 1, 2 and 3, respectively.

Figure S3.4: Exponential curve fitting between soil temperature and root derived respiration (R_r_) and R_h_ for the three models.

The partitioning of the different R_s_ components using sequential sampling dates allowed us to estimate the seasonal trends in the root contribution to R_s_ (Fig. S3.4).

***S4: DOC Leaching***

Figure S4.1: Course of the monthly precipitation, evapotranspiration and water balance during the four years of the two rotations.

Figure S4.2: Water balance and DOC concentration in the soil during the soil water sampling campaigns from August 2011 to July 2013. The dashed line represents the monthly water balance of the measured precipitation minus evapotranspiration. The squares represent the monthly average of DOC, and the black line shows the average DOC = 23 g C m^-3^. DOC= dissolved organic carbon (modified after M. Camino Serrano).

**S5: SOC balance using the eddy-covariance+biometric approach**

SOC balance was estimated indirectly using eddy-covariance and biometric measurements.

Eddy covariance measurements:

The eddy covariance (EC) system used to measure gas and energy fluxes between the SRC plantation and the atmosphere included the following: a three-dimensional sonic anemometer

(model CSAT3, Campbell Scientific, Logan, UT, USA) to measure the wind speed components, a closed-path differential infrared gas analyzer (LI-7000, LI-COR, Lincoln, NE, USA) for the measurements of the CO_2_ and H_2_O mole fractions in the air, a closed-path fast-response N_2_O/CO analyzer (model 908-0014, Los Gatos Research, Mountain View, CA, USA) and a closed-path fast-response CH_4_ analyzer (model DLT-100, Los Gatos Research, Mountain View, CA, USA). Measurements were made continuously with all the instruments sampling at a frequency of 10 Hz from June 1, 2010 to December 31, 2013.

SOC balance:

ΔSOC = NECB –ΔB [Eq. S5.1]

In most ecosystems the C exchange is driven by vertical C fluxes as photosynthesis and respiration; but in agroecosystems horizontal C fluxes as anthropogenic C removal (harvest of C) play a very important role.

Net ecosystem CO_2_ exchange (NEE), measured using eddy-covariance methods, integrates vegetation and soil CO_2_ fluxes, and provides a measure of net carbon exchange. The net ecosystem carbon balance (NECB) represents the overall ecosystem C balance from all sources and sinks*, i.e.* physical, biological, and anthropogenic:

NECB = dC/dt [Eq. S5.2]

Net fluxes of several forms of C contribute to NECB:

NECB= -NEE + FCH_4_ + Fvoc + Fdoc + Fpc [Eq. S5.3]

where NEE is net ecosystem exchange (*i.e.* the net C0_2_ flux from the ecosystem to the atmosphere); FCH_4_ is the net methane (CH_4_) efflux; FVOC is the net volatile organic C (VOC) efflux; FDOC is the net dissolved organic C (DOC) leaching loss; and FPC is the net lateral transfer of C out of the ecosystem by processes such as anthropogenic transport or harvest.

NEE and CH_4_ efflux data were obtained from Zenone et al. (2015), using the sign convention that negative fluxes represent an uptake by the SRC plantation.

Table S5.1: Fluxes of C measured with eddy-covariance technique.

|  | NEE | | CH_4_ efflux | |
| --- | --- | --- | --- | --- |
|  | g C m^-2^ | SD | g C m^-2^ | SD |
| 2010 | 75.2 | 4.4 | 17.2 | 1.4 |
| 2011 | -95.6 | 5.9 | 13.4 | 1.4 |
| 2012 | 151 | 10.5 | 27.2 | 1.4 |
| 2013 | -274.6 | 18.8 | 35.4 | 25.1 |
| 2010-2013 | -144 | 22.8 | 93.2 | 25.2 |

VOC flux data were obtained from Brilli et al (2014). VOC emissions were rather low in terms of C flux, being isoprene the most abundant VOCs emitted. Its exchange with the atmosphere displayed a trend whit no emissions in winter, low emissions in cold and cloudy periods (0.023 g C m^-2^ d^-1^) and peaks in summer with sunny and warm periods (0.117 g C m^-2^ d^-1^). Considering a winter season of four months, and half cloudy/ half sunny days in the remaining eight months –as typical for Belgium- a total of 0.68 Mg C ha^-1^ was estimated for the four years.

The FPC or harvested biomass data were obtained from Verlinden et al (2015). The overall harvested biomass was 32.4 Mg DM ha^-1^ with a C% of 44.6 ±0.29.

***S6: Error estimation and Uncertainty analysis***

The uncertainty analysis was done following the *IPCC Good Practice Guidance and Uncertainty Management in National Greenhouse Gas Inventories (IPCC 2006).*

For the purpose of propagating uncertainties in the C balance, we used two general methods: the approximation method based on a first order Taylor series expansion, often referred to as the error propagation formulas, and the numerical Monte Carlo method. The simple error propagation formulas were used in the early stages of the calculation, until an input value was obtained (Figure S6.1). The combination of these uncertainties resulted in an estimation of DM input uncertainty at single variable level for each year.


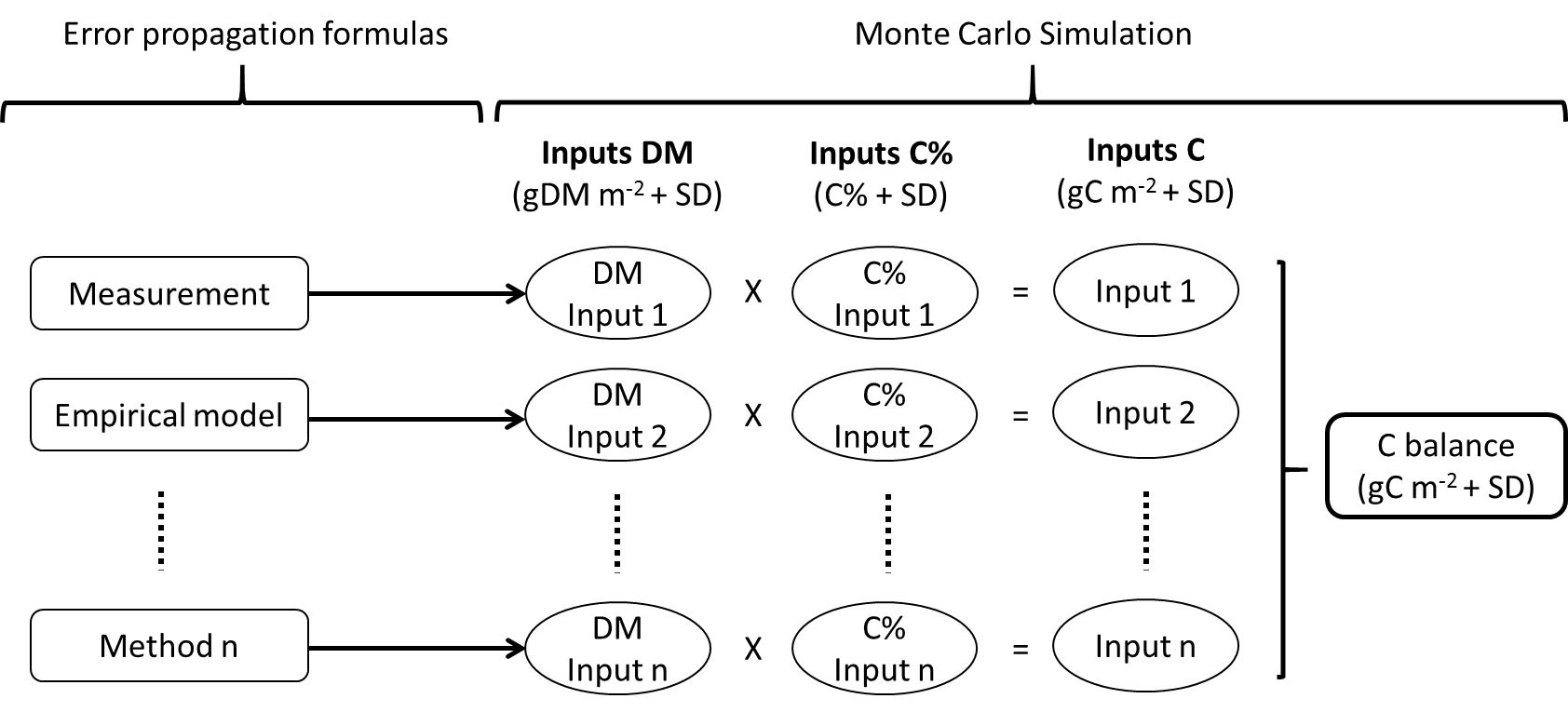


Figure S6.1: Representation of uncertainty analysis showing the use of error propagation formulas on early calculations and Monte Carlo Simulations at the last stages.

Once an input value was obtained per variable and per year, the calculation was continued using the most widely used numerical technique for the problem, the Monte Carlo simulation (MCS). For each variable a probability function was chosen. As we worked with empirical data, it was assumed they had a normal distribution. Other distributions were used only where there were compelling reasons, either from empirical observations or from expert judgement backed up by theoretical argument; for example truncated normal distribution to avoid negative values. The run of MCS included stochastic sampling of 1000 iterations on each DM input and C% for each input variable.

**S7: Models used for statistics**

The software InfoStat (Di Rienzo *et al*., 2011) was used for the analysis.

ANOVA: C concentration (%) differences between genotypes (Table 1 of the manuscript)

We performed one model for each soil layer.

year Depth Variable N R² R² Aj CV

2014.00 A C 40 0.01 0.00 16.02

**(SC type III)**

V SqS fd MS F p-value

Model 0.01 1 0.01 0.20 0.6609

Genotype 0.01 1 0.01 0.20 0.6609

Error 2.52 38 0.07

Total 2.53 39

year Depth Variable N R² R² Aj CV

2014.00 B C 40 0.05 0.02 24.47

**(SC type III)**

V SqS fd MS F p-value

Model 0.32 1 0.32 1.86 0.1804

Genotype 0.32 1 0.32 1.86 0.1804

Error 6.57 38 0.17

Total 6.90 39

year Depth Variable N R² R² Aj CV

2014.00 C C 40 0.03 0.00 31.83

**(SC type III)**

V SqS fd MS F p-value

Model 0.20 1 0.20 0.99 0.3272

Genotype 0.20 1 0.20 0.99 0.3272

Error 7.56 38 0.20

Total 7.75 39

year Depth Variable N R² R² Aj CV

2014.00 D C 40 0.03 2.1E-03 69.73

**(SC type III)**

V SqS fd MS F p-value

Model 0.81 1 0.81 1.08 0.3044

Clone 0.81 1 0.81 1.08 0.3044

Error 28.27 38 0.74

Total 29.08 39

ANOVA: SOC in equivalent soil mass (Table 2 of the manuscript)

We performed one model for each soil layer. Land: former land-use; year: comparisons between 2010 and 2014; Genotypes: Skado and Koster.

Depth Variable N R² R² Aj CV

A SOCeq 128 0.37 0.33 14.28

**(SC type III)**

V SqS fd MS F p-value

Model 16.21 7 2.32 9.90 <0.0001

Land 3.96 1 3.96 16.93 0.0001

year 1.89 1 1.89 8.07 0.0053

Genotype 0.31 1 0.31 1.31 0.2550

Land*year 4.10 1 4.10 17.54 0.0001

Land*Genotype 0.14 1 0.14 0.60 0.4386

year*Genotype 0.06 1 0.06 0.27 0.6068

Land*year*Genotype 0.12 1 0.12 0.51 0.4771

Error 28.07 120 0.23

Total 44.27 127

Depth Variable N R² R² Aj CV

B SOCeq 128 0.42 0.39 23.45

**(SC type III)**

V SqS fd MS F p-value

Model 47.49 7 6.78 12.42 <0.0001

Land 0.12 1 0.12 0.22 0.6425

year 24.58 1 24.58 45.01 <0.0001

Genotype 0.14 1 0.14 0.25 0.6162

Land*year 15.26 1 15.26 27.95 <0.0001

Land*Genotype 0.92 1 0.92 1.69 0.1967

year*Genotype 4.70 1 4.70 8.61 0.0040

Land*year*Genotype 0.79 1 0.79 1.45 0.2316

Error 65.53 120 0.55

Total 113.02 127

Depth Variable N R² R² Aj CV

C SOCeq 128 0.11 0.06 33.42

**(SC type III)**

V SqS fd MS F p-value

Model 17.11 7 2.44 2.14 0.0440

Land 1.09 1 1.09 0.96 0.3304

year 4.53 1 4.53 3.98 0.0484

Genotype 0.65 1 0.65 0.57 0.4530

Land*year 0.14 1 0.14 0.12 0.7283

Land*Genotype 6.43 1 6.43 5.64 0.0191

year*Genotype 1.13 1 1.13 0.99 0.3208

Land*year*Genotype 0.20 1 0.20 0.18 0.6763

Error 136.76 120 1.14

Total 153.87 127

Depth Variable N R² R² Aj CV

D SOCeq 128 0.38 0.35 63.25

**(SC type III)**

V SqS fd MS F p-value

Model 184.16 7 26.31 10.58 <0.0001

Land 16.61 1 16.61 6.68 0.0110

year 110.22 1 110.22 44.33 <0.0001

Genotype 6.58 1 6.58 2.65 0.1064

Land*year 2.44 1 2.44 0.98 0.3239

Land*Genotype 37.10 1 37.10 14.92 0.0002

year*Gentype 2.83 1 2.83 1.14 0.2885

Land*year*Genotype 3.61 1 3.61 1.45 0.2308

Error 298.36 120 2.49

Total 482.52 127

**S8: Soil C in control plots**

There were no differences in the soil C concentrations (%) at any depth in the control plots over the four-years study period.

Table S7.1: Vertical distribution of soil C% in the control grasslands for years 2010 (previous the conversion) and 2014. n=8; p<0.01.
